# Outbreak of Legionnaire’s Disease Caused by *Legionella pneumophila* Serogroups 1 and 13 

**DOI:** 10.3201/eid2302.161012

**Published:** 2017-02

**Authors:** Toshiro Kuroki, Junko Amemura-Maekawa, Hitomi Ohya, Ichiro Furukawa, Miyuki Suzuki, Tomoka Masaoka, Kastuhiro Aikawa, Kazumi Hibi, Masatomo Morita, Ken-ichi Lee, Makoto Ohnishi, Fumiaki Kura

**Affiliations:** Kanagawa Prefectural Institute of Public Health, Kanagawa, Japan (T. Kuroki, H. Ohya, I. Furukawa, M. Suzuki, T. Masaoka, K. Aikawa, K. Hibi);; National Institute of Infectious Diseases, Tokyo, Japan (J. Amemura-Maekawa, M. Morita, K. Lee, M. Ohnishi, F. Kura)

**Keywords:** *Legionella pneumophila*, serogroup, outbreak, pneumonia, spa house, dual infection, bacteria, Japan, Legionnaire’s disease

## Abstract

In Japan, hot springs and public baths are the major sources of legionellosis. In 2015, an outbreak of Legionnaires’ disease occurred among 7 patients who had visited a spa house. Laboratory investigation indicated that *L. pneumophila* serogroup 1 and 13 strains caused the outbreak and that these strains were genetically related.

Infection with *Legionella* bacteria is one of the major causes of community-acquired pneumonia (*1*). In Japan, the major sources of *Legionella* infection are hot springs and public baths (*2*). Among *Legionella* species, *L. pneumophila* serogroup 1 accounts for most human infections (*3*). Legionellosis outbreaks caused by a combination of *L. pneumophila* serogroup 1 or other serogroups have rarely been reported. We report an outbreak of Legionnaires’ disease caused by *L. pneumophila* strains of serogroup 1 and serogroup 13.

During June 1–17, 2015, the Health Centers in Kanagawa and Shizuoka Prefectures, Japan, were notified of 7 cases of *Legionella* pneumonia. All patients with pneumonia were admitted to 1 of 5 hospitals. All patients were male (mean age 66.3 years), 4 had diabetes mellitus, and 1 had hepatic cirrhosis and liver cancer. Diagnosis of pneumonia at the hospitals was based on clinical presentation and immunochromatographic detection of *L. pneumophila* serogroup 1 antigen in urine. All patients recovered and were discharged.

In epidemiologic interviews, all 7 patients stated that they had visited a spa house in Odawara, Kanagawa, Japan, before illness onset. The latent period was not accurately determined because 5 of the 7 patients frequently visited this spa house and some patients had visited it again after illness onset. The spa house had 7 circulating systems, including filtration and heating components, and 9 baths for men. 

Sputum samples from 5 urinary antigen–positive patients and environmental samples from the spa house were collected for epidemiologic investigations and cultured for *Legionella* at the Kanagawa Prefectural Institute of Public Health. *L. pneumophila* serogroup 1 was detected in sputum from 4 patients (Table), and *L. pneumophila* serogroups 1 and 13 were detected in sputum from 1 patient (patient 2). Baths 1–5, but not baths 6–9, contained *L. pneumophila*. Epidemiologic investigation and laboratory results revealed that failure to adequately chlorinate the bath water and the circulating systems resulted in colonization of *Legionella* at the spa house.

**Table:** Genetic characteristics of *Legionella pneumophila* isolates from patients with pneumonia and from environmental samples, Japan, 2015*

Serogroup	PGFE profile†	ST‡	ST profile, *flaA, pilE, asd, mip, mompS, proA, neuA*	Sample source	cgMLST profile§
1	A	ST2114	6, 10, 21, 3, 17, 14, 9	Patients 1, 7	a, b
				Bath 1 (bath water)¶	b
				Bath 2 (spout swab)	b
1	A	ST2121	6, 10, 21, 3, 17, 14, 57	Bath 1 (bath water)¶	a
1	B	ST2114	6, 10, 21, 3, 17, 14, 9	Patients 2, 5, 7	b, b, b
				Bath 1(bathtub swab)	b
				Bath 2 (bath water)¶	b
1	C	ST1447	6, 10, 20, 13, 9, 4, 11	Bath 3 (hair trap debris)	c
13	B	ST2113	6, 10, 21, 10, 17, 14, 209	Patient 2	d
				Bath 1 (bath water)¶	d
				Bath 2 (bath water)¶	Not done
				Bath 2 (spout swab)	d
10	D	ST2115	7, 10, 17, 3, 13, 14, 207	Bath 4 (spout swab)	e
				Bath 5 (bathtub swab)	f

*cgMLST, core genome multilocus sequence typing; PFGE, pulsed-field gel electrophoresis; ST, sequence type. †Profiles A and B were obtained from clinical and environmental samples. Profiles C and D were obtained from environmental samples only.  ‡New STs from this study were assigned ST2113, ST2114, ST2115, and ST2121. §Each profile letter indicates a tentative cgMLST profile of 1 strain. ¶Concentrations of *L. pneumophila* in bath water were 800 CFU/L in bath 1, and 1,100 CFU/L in bath 2.

Pulsed-field gel electrophoresis (PFGE) comparison (*4*) of clinical and environmental isolates revealed that the *L. pneumophila* serogroup1 strains produced 3 PFGE profiles: A and B, with a 1-band difference, and C (Table). Patient 7 was infected with 2 *L. pneumophila* serogroup 1 isolates possessing PFGE profiles A and B. The PFGE profile B of *L. pneumophila* serogroup 13 was identical to that of serogroup 1. We determined the sequence type (ST) of *L. pneumophila* strains (*5*,*6*) and identified 4 new STs: ST2113, ST2114, ST2115, and ST2121. ST2114 differed from ST2121 by only 1 nt in *neuA* and differed from ST2113 (serogroup 13) by only 2 alleles (*mip* and *neuA*), suggesting that these 3 STs are closely related and that 1 of 3 strains (ST2113, ST2114, or ST2121) may be derived from another by homologous recombination. All examined isolates lacked the *lag-1* gene, a virulence-associated marker (*7*).

By using whole-genome sequencing, we applied core-genome multilocus sequence typing (cgMLST) with 50 genes (*8*) different from the 7 genes in sequence-based typing, thereby confirming the sequence-based typing data (Table). ST2114 and ST2115 isolates were each divided into 2 cgMLST profiles. cgMLST profile b differed from profile a by only 1 nt on *lpg1503* and differed from profile d by 5 nt on *lpg0812*, near the lipopolysaccharide coding region, suggesting that the strain of profile d may be derived from another by homologous recombination. However, the remaining cgMLST profiles (c, e, and f) from strains not isolated from patients differed from profile b by 30, 42, and 43 alleles, respectively.

Multiple *L. pneumophila* strains with different genetic characteristics exist in the environment and pose infection risks (*9*). Among the strains studied, dual infections with *L. pneumophila* serogroup 1 and serogroup 13 strains (patient 2) and *L. pneumophila* serogroup 1 strains with different genomic subtypes (patient 7) were detected. Results from 3 genetic methods revealed that *L. pneumophila* serogroup 1 and 13 strains are closely related, although the serogroups differ. Results of this study were consistent with the hypothesis that multiple infections are more likely with less virulent strains and more likely in persons with medical conditions predisposing them to Legionnaires’ disease (*10*). 

Our study of this outbreak suggests that the spa house was colonized by several *L. pneumophila* strains that were genetically related despite belonging to different serogroups and that 2 strains caused infection. Further analysis of the divergence of outbreak strains in genomes related to *Legionella* serogroup and sequence types is ongoing. This analysis clarifies the in-depth genetic relations among *L. pneumophila* strains, such as recombination sites and periods required for divergence. We recommend that the spa house provide high quality management and effective infection control practices according to an infection control manual (e.g., completion of documentation relating to infection control practices and training of employees) and that customers be aware of the sanitary status of spa houses.
